# G Protein-Coupled Receptor 109A Maintains the Intestinal Integrity and Protects Against ETEC Mucosal Infection by Promoting IgA Secretion

**DOI:** 10.3389/fimmu.2020.583652

**Published:** 2021-01-08

**Authors:** Yuhong Gong, Xinxin Jin, Boyu Yuan, Yantao Lv, Guangmou Yan, Mingming Liu, Changxin Xie, Juxiong Liu, Yimei Tang, Hongyan Gao, Yufeng Zhu, Yanhua Huang, Wei Wang

**Affiliations:** ^1^ Innovative Institute of Animal Healthy Breeding, College of Animal Science & Technology, Zhongkai University of Agriculture and Engineering, Guangzhou, China; ^2^ Laboratory Animal Center of Nanfang Hospital, Southern Medical University, Guangzhou, China; ^3^ Key Laboratory of Zoonosis Research, Ministry of Education, Jilin University, Changchun, China; ^4^ Department of Pharmacology, College of Basic Medical Science, Jilin University, Changchun, China; ^5^ College of Veterinary Medicine, Jilin University, Changchun, China

**Keywords:** GPR109A, enterotoxigenic *Escherichia coli*, secretory IgA, epithelium barrier, sodium butyrate

## Abstract

Several studies have reported an intricate link between the G protein-coupled receptor 109A (GPR109A) and intestinal health. Upon activation, induced by butyric acid and β-hydroxybutyric acid, GPR109A regulates the expression of tight junction proteins, exerts anti-inflammatory effects, and maintains the integrity of the intestinal barrier. However, its function and the mechanism of action in combating the infection caused by exogenous pathogenic microorganisms remain unclear. This study established an animal model of infection by oral enterotoxigenic *Escherichia coli* (ETEC) gavage to examine the underlying mechanism(s) and protective effects of GPR109A on the intestinal tract. Experimental GPR109A^–/–^and GPR109A^+/+^ mice were orally administered with 1 × 10^9^ colony-forming units (CFUs) of ETEC, and changes in body weight were then observed. The colonization and translocation of ETEC in the intestine were detected by the plate counting method. The expression of tight junction proteins and the levels of inflammatory factors and secretory IgA (SIgA) in the intestine were detected by quantitative real-time polymerase chain reaction (q-PCR), western blotting, enzyme-linked immunosorbent assay (ELISA), and immunohistochemistry. The results demonstrated that GPR109A^–/–^mice were more susceptible to ETEC infection, showing more severe inflammatory reactions and intestinal damage. Moreover, the secretion of IgA in the intestinal tract of GPR109A^+/+^ mice was significantly increased after ETEC infection, whereas the IgA levels in GPR109A^–/–^mice did not change significantly. We added 5 g/L sodium butyrate to the drinking water of all mice. The GPR109A^+/+^ mice were protected against ETEC infection and no effect was observed in GPR109A^–/–^mice. Similarly, sodium butyrate increased the SIgA content in the gut of the GPR109A^+/+^ mice and no effect was observed in GPR109A^–/–^mice. In conclusion, activated GPR109A is effective against the colonization and translocation of ETEC in the gut and maintains the integrity of the intestinal barrier, possibly by promoting the secretion of intestinal IgA.

## Introduction

The intestine is the largest organ involved in digestion and absorption in animals and the largest immune and endocrine organ. It plays a crucial role in nutrient absorption, preventing pathogens from invading the body and intestinal microbial infections, thereby acting as a major barrier of defense. The integrity of the intestinal mucosal epithelium is essential for maintaining homeostasis (1, 2). Therefore, understanding the mechanisms regulating this barrier is essential for preventing microbial infection and predicting disease progression.

Enterotoxigenic *Escherichia coli* (ETEC) is a gram-negative ornithobacterium that belongs to the genus *Escherichia* of the family Enterobacteriaceae. The enterotoxins produced by ETEC can be divided into heat-stable enterotoxins (STs) and heat-labile enterotoxin (LTs) (3, 4) depending on their functions in pathology and immunology. Enterotoxin, a proteinaceous toxin, has strong pathogenicity for the digestive tract in youngstock and is responsible for causing diarrhea in young animals. It activates guanine cyclase in the intestinal epithelial cells (IECs), affects electrolyte production, and causes diarrhea (5). Simultaneously, endothelial cells are activated, releasing cytokines and inflammatory mediators, leading to increased inflammatory response and damaging local tissues (6–8). ETEC travels through the damaged intestinal epithelium to the mesenteric lymph nodes (MLNs), spreading to the spleen, liver, and other organs for colonization.

G protein-coupled receptor 109A (GPR109A, HM74A in humans) was first demonstrated to promote the function of niacin in lowering blood lipids (9). Recent studies have reported that GPR109A can significantly inhibit the inflammatory response in different diseases such as Alzheimer’s Disease, mastitis, sepsis, obesity, diabetes, and colitis, following activation by its ligands (10). After GPR109A is activated by short-chain fatty acids, it can regulate the activation of intestinal inflammasome NLRP3 and regulate local inflammation (11). Furthermore, we have previously shown that the activation of GPR109A by β-hydroxybutyrate or butyrate significantly reduced the secretion of inflammatory cytokines by inhibiting lipopolysaccharide (LPS)-induced NF-κB signaling pathways in microglia and peripheral macrophages (12, 13). In addition, we reported significant differences in the intestinal flora between GPR109A^–/–^and GPR109A^+/+^ mice under the same feeding conditions. The proportion of *Firmicutes*, *Proteobacteria*, and *Verrucomicrobia* was lower in the feces of GPR109A^+/+^ mice as compared to that in GPR109A^–/–^mice (14). Pathogenic bacteria, such as *E. coli* and *Salmonella*, which cause intestinal inflammation and diarrhea, belong to Proteobacteria, suggesting that GPR109A limits the colonization and proliferation of harmful intestinal bacteria in the digestive tract.

We studied the protective effects of GPR109A on intestinal health during the infection by ETEC. Our data suggested that GPR109A protected the mice from ETEC infection, promoted the expression of tight junction proteins, and reduced the levels of proinflammatory cytokines to maintain intestinal homeostasis. Moreover, we found that GPR109A increased the secretion of intestinal IgA, which resists the submucosal colonization of foreign pathogens in the intestinal tract.

## Materials and Methods

### Animals

Male GPR109A^+/+^ and GPR109A^–/–^mice born in the same litter, 5 to 7-weeks old, were used. The GPR109A^–/–^mice were donated by Dr. Martin Sager and were subsequently hybridized with C57BL/6J mice (14). The mice were housed in a free-feeding environment at 22 to 23°C with a natural light cycle.

### Inoculation of Mice *In Vivo*

The ETEC infection model was established in mice using the method described by Allen (15). In brief, all mice were administered streptomycin (5 g/L)-containing water for 72 h before being infected with ETEC to eradicate the native flora in the gut. The mice were starved for 12 h before inoculation, and streptomycin-treated water was replaced with sterile water 12 h before ETEC inoculation. The treated mice were orally inoculated (gavage) with 1 × 10^9^ colony-forming units (CFUs) of the ETEC strain K88, diluted with 0.1 M carbonate buffer (pH 9.0) (7). The control group was orally administered an equal volume of sterile phosphate buffer (phosphate-buffered saline [PBS]). As reported in other studies (13), the mice received sodium butyrate treatment one day before the supplementation of streptomycin. Sodium butyrate (5 g/L) was added to drinking water and continued throughout the experiment (16). Five days after the infection, the mice were sacrificed to collect the jejunum, ileum, spleen, liver, and mesenteric lymph nodes (MLNs). A part of the collected tissues was homogenized and subsequently plated on the kanamycin-resistant MAC agar medium. Another part of the tissue was stored in a −80°C ultra-low temperature refrigerator.

### Quantitative Real-Time PCR (RT-PCR, q-PCR)

The total RNA was extracted, reverse transcribed, and subjected to RT-PCR as per our previously described method (17). Briefly, the cryopreserved jejunum and colon tissues were pulverized with a cell/tissue grinder. RNAiso Plus (TaKaRa, 9108) was used to extract the total RNA according to the manufacturer’s instructions. A commercial reverse transcription kit (TaKaRa, RR036A) was used to generate the cDNA. A reaction mixture (20 µL) of cDNA, primers, and SYBR Green (TaKaRa, RR820A), was subjected to RT-PCR to evaluate the mRNA levels of TNF-α, IL-1β, IL-6, Cldn1, Cldn2, Cldn3, Ocln, Zo-1, Zo-2, PIGR, and J-chain. The primer sequences used in this study are listed in **Table 1**.

**Table 1:** Primers for real-time PCR.

Gene	Sequences	Length (bp)
IL-1β	Forward: 5′- TGTGATGTTCCCATTAGAC–3′	87
	Reverse: 5′- AATACCACTTGTTGGCTTA–3′	
IL-6	Forward: 5′- AGCCACTGCCTTCCCTAC–3′	133
	Reverse: 5′- TTGCCATTGCACAACTCTT–3′	
TNF-α	Forward: 5′- CCACGCTCTTCTGTCTACTG–3′	110
	Reverse: 5′- GCTACGGGCTTGTCACTC–3′	
GPR109A	Forward: 5′-CCGTCGTGTAGTCTGTCTCGTG-3′	119
	Reverse: 5′-GCTGCGGTTATTGTTGGACT-3′	
Cldn1	Forward: 5′- GGTGCCTGGAAGATGATGAGGTG–3′	91
	Reverse: 5′- GCCACTAATGTCGCCAGACCTG–3′	
Cldn2	Forward: 5′- AGTGGCTGTAGTGGGTGGAG–3′	197
	Reverse: 5′-AAAGGATGACTCCGGCTACC–3′	
Cldn3	Forward: 5′- CTA CCG GCC AGA GTA TGC AG–3′	183
	Reverse: 5′- TTG CGG CAA TGA AAG GCA TC–3′	
GAPDH	Forward: 5′-GCCATCACTGCCACCCAGAA-3′	153
	Reverse: 5′-GCCAGTGAGCTTCCCGTTGA-3′	
Zo-1	Forward: 5′-GACCTTGATTTG CATGACGA-3′	199
	Reverse: 5′-AGGACCGTGTAATGGCAGAC-3′	
Zo-2	Forward: 5′-CAGTCCCTATGCCTGAGAGC-3′	201
	Reverse: 5′-TTG GAA CCG CAT AGA TGT CA-3′	
Ocln	Forward: 5′-ACA CTT GCT TGG GAC AGA GG-3′	197
	Reverse: 5′-AAG GAA GCG ATG AAG CAG AA-3′	
PIGR	Forward: 5′-CGAGGATGCTGGCTTCTATTGGTG-3′	119
	Reverse: 5′-CGTTCTGTGGCGTCACCTCAAG-3′	
J-chain	Forward: 5′-AATGCGATCCTGTGGAAGTGGAG-3′	101
	Reverse: 5′-CATGTAGCAGGTCTCAGGAACACC-3′	

### Enzyme-Linked Immunosorbent Assay

Enzyme-linked immunosorbent assay (ELISA) was carried out using kits (MEINIAN, MM-0430M2). The mouse jejunum tissue was washed with cold PBS, weighed, and homogenized. The homogenate was diluted in a proportion of 2 mL of cold PBS per gram of the tissue. After centrifuging the homogenate, the supernatant was collected to analyze SIgA according to the manufacturer’s instructions.

### Western Blotting

The mouse jejunum was homogenized in P0013B containing a phosphatase inhibitor and protease inhibitor. The homogenate was allowed to stand at 4°C for 40 min to facilitate the lysis. The lysate was centrifuged for 20 min, and the supernatant was collected as the total protein for immunoblotting. Subsequently, western blotting was performed according to the standard protocol (17). Antibodies against β-tubulin (1:4000; Proteintech, 10094–1-AP), Cldn1 (1:2000; Abcam, ab15091), Cldn3 (1:2000; Abcam, ab15102), Ocln (1:2000; Abcam, ab31721), goat anti-rabbit IgG-horse radish peroxidase (HRP), and goat anti-mouse IgG-horse radish peroxidase (HRP; 1:3000, Santa Cruz Biotechnology, Sc-2004/5) were used.

### RNA Interference and Overexpression

Lipofectamine 2000 (Invitrogen, Carlsbad, CA) was used to overexpress GPR109A. Caco-2 cells were stably transfected for 48 h with plasmid pcDNA3.0 encoding human GPR109A. A lentiviral interference vector of GPR109A was synthesized by Shanghai Sangon Biological Engineering Technology. The infection was performed following the manufacturer’s protocol. Briefly, Caco-2 cells were grown overnight in 6-well plates (2 × 10^5^ cells/well), infected with HCV (multiplicity of infection [MOI] 20), and incubated for 24 h. The success of lentivirus infection was assured by monitoring the green fluorescent protein (GFP)-positive cells under a fluorescence microscope, and the cells were used for subsequent experiments.

### Plasmid Construction, Extraction, and Transformation

The GFP gene sequence was inserted into the PET-28a vector, which was expressed in the *E. coli* BL21 strain. The plasmid was extracted using the TIANprep Mini Plasmid kit (TIANGEN, DP103) according to the manufacturer’s instructions. The DNA was transfected into the ETEC strain K88 at 1800 V by an electroporator. The cells were transferred to a suitable culture tube and incubated for 1 h at 37°C. A suitable volume of the bacterial solution was applied to a kanamycin-resistant agar plate and cultured at 37°C overnight to observe the results.

### Hematoxylin and Eosin Staining and Immunohistochemistry

The colon and ileum segments with a length of 1 to 2 cm were cut, washed in cold PBS, fixed in 4% formaldehyde for 24 h, and used for paraffin sectioning, with a section thickness of 5 µm. A part of the paraffin section was used for hematoxylin and eosin (H&E) staining, and the other section was used for immunohistochemical detection according to the previously described procedure (12). In brief, the tissue sections were boiled in sodium citrate buffer to completely expose the antigen sites. After cooling to room temperature, the paraffin section was washed thrice with PBS, treated with 50 µL of peroxidase blocker for 10 min, and washed again. The sections were blocked with 0.5% goat serum for 1 h at room temperature, and the excess serum was absorbed by a filter paper without washing. Next, the sections were incubated with anti-IgA (1:200; Santa Cruz Biotechnology, Sc-373823) and anti-PIGR (1:200, Santa Cruz Biotechnology, Sc-374343) antibodies for 12 h at 4°C. The sections were washed and incubated for 10 min at room temperature with biotin-labeled secondary antibodies (Biozol; Eching, Germany) at a dose of 50 µL per slice. The avidin–peroxidase solution was incubated for 10 min at room temperature with a 50 µL per slice dose, washed with PBS for 5 min, stained with 3,3’-diaminobenzidine (DAB; IBL, Germany), washed again, and dehydrated.

### RNA Sequencing and Data Analysis

Sodium butyrate (2 mM) was used to treat Caco-2 cells for 12 h, following which total RNA was extracted. RNA samples were isolated using RNAiso Plus (TaKaRa, 9108). After extracting the total RNA, mRNA was enriched using magnetic beads with Oligo(dT). Further, the fragmentation buffer was added to generate short fragments. The fragmented mRNA was used as a template to synthesize the first strand of cDNA. Subsequently, the buffer, dNTPs, RNase H, and DNA polymerase I was added to synthesize the second strand of cDNA. The target fragments were recovered by agarose gel electrophoresis and amplified by PCR. The constructed library was sequenced with Illumina HiSeq2000. Genes with *p*-values < 0.05 and fold change > 2 were considered to be differentially expressed.

### Statistical Analysis

The results are expressed as mean ± standard deviation (SD). Except for the RNA sequence analysis, all experiments were performed three times with similar results. Statistical analysis was performed using Student’s *t*-test or one-way analysis of variance (ANOVA) and analyzed using GraphPad Prism 7.0. A *p*-value < 0.05 was considered statistically significant.

## Results

### GPR109A Is Critical in Limiting ETEC Colitis

**Figure 1:**
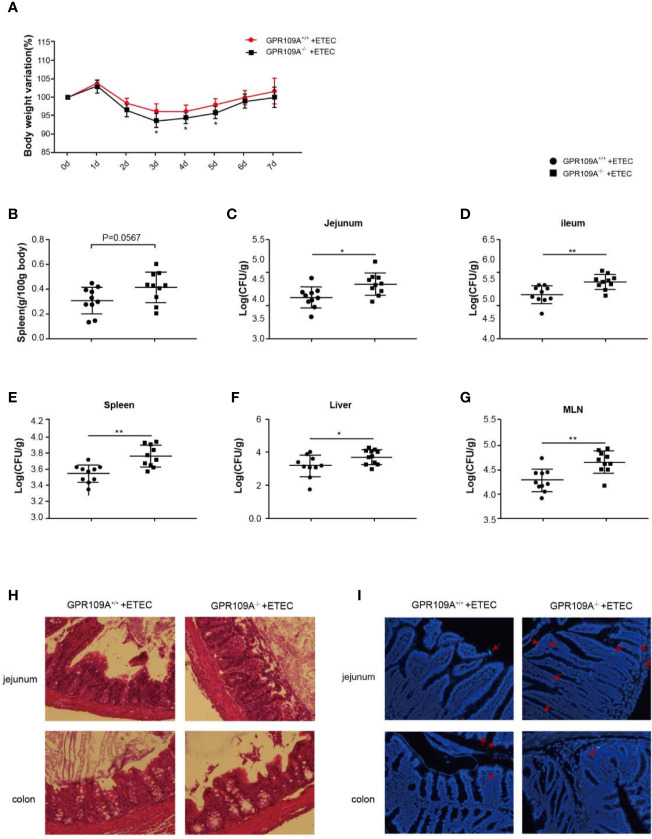
GPR109A deficiency increases the susceptibility to oral ETEC infection. GPR109A^+/+^ and GPR109A^–/–^ mice were treated with streptomycin before the oral administration of 1 × 10^9^ colony-forming units (CFUs) of ETEC. Some mice were used to observe the change in body weight, and others were sacrificed after five days of infection to test the bacterial load. **(A)** Body weight loss in the ETEC-treated GPR109A^+/+^ and GPR109A^–/–^mice. 0–5 d: *n* = 14, 5–7 d: *n* = 4. * represent significant difference compared with 0d. **(B)** Spleen weight, *n* = 10. **(C–G)** Bacterial load in the jejunum, ileum, spleen, liver, and mesenteric lymph nodes measured by plate counting method, *n* = 10. **(H)** Representative images of hematoxylin and eosin (H&E) staining of the jejunum and colon, magnification ×40. **(I)** Representative images of the colonization and translocation of ETEC in the jejunum and colon, magnification ×40. The red arrow indicates GFP-labeled ETEC.The values are expressed as mean ± standard deviation (SD), ^*^ *p* < 0.05, ^**^ *p* > 0.01. The presented data are the average of three independent experiments.

Some recent studies have reported a close link between intestinal flora and endocrine diseases, cardiovascular diseases, digestive diseases, tumors, and diseases of the central nervous system (18, 19). The abundance and composition of the intestinal microbiota are regulated by several factors such as diet, age, drugs, and genotype (20). The differences in the composition of fecal flora in GPR109A^–/–^and GPR109A^+/+^ mice under the same feeding conditions were determined by 16S rRNA sequencing. It was observed that GPR109A altered the relative abundance of the intestinal flora of mice (14). To further investigate how GPR109A affected the host flora structure, we examined the host response to evaluate the involvement of GPR109A in ETEC infection. After establishing an infection model using the ETEC strain K88, which was bioengineered to express GFP, a weight loss was observed in GPR109A^+/+^ and GPR109A^–/–^mice at the same time each day. There was no significant difference in the weight loss between GPR109A^+/+^ and GPR109A^–/–^ mice. Nevertheless, a comparison of body weight for seven days showed that GPR109A-deficient mice had low body weight variations from day 3 to day 5 after ETEC gavage (**Figure 1A**). The spleen weights were examined to further evaluate the spread of ETEC. The GPR109A^–/–^mice exhibited more severe splenomegaly (p = 0.0567) (**Figure 1B**). The colonization and translocation of ETEC were detected in the mice. An increase in the the bacterial load in the jejunum, ileum, spleen, liver, and MLNs of GPR109A-deficient mice was observed compared to in GPR109A^+/+^ mice (**Figures 1C–G**), indicating the higher bacterial transmission in GPR109A^–/–^mice. H&E staining showed that GPR109A^–/–^mice had more severe intestinal structural damage compared to the GPR109A^+/+^ mice (**Figure 1H**). **Figure 1I** shows the colonization and translocation of ETEC in the submucous layer of the intestine. These results indicate that the absence of GPR109A exacerbated the body’s susceptibility to ETEC.

### GPR109A Maintains Intestinal Tissue Homeostasis and Integrity

**Figure 2:**
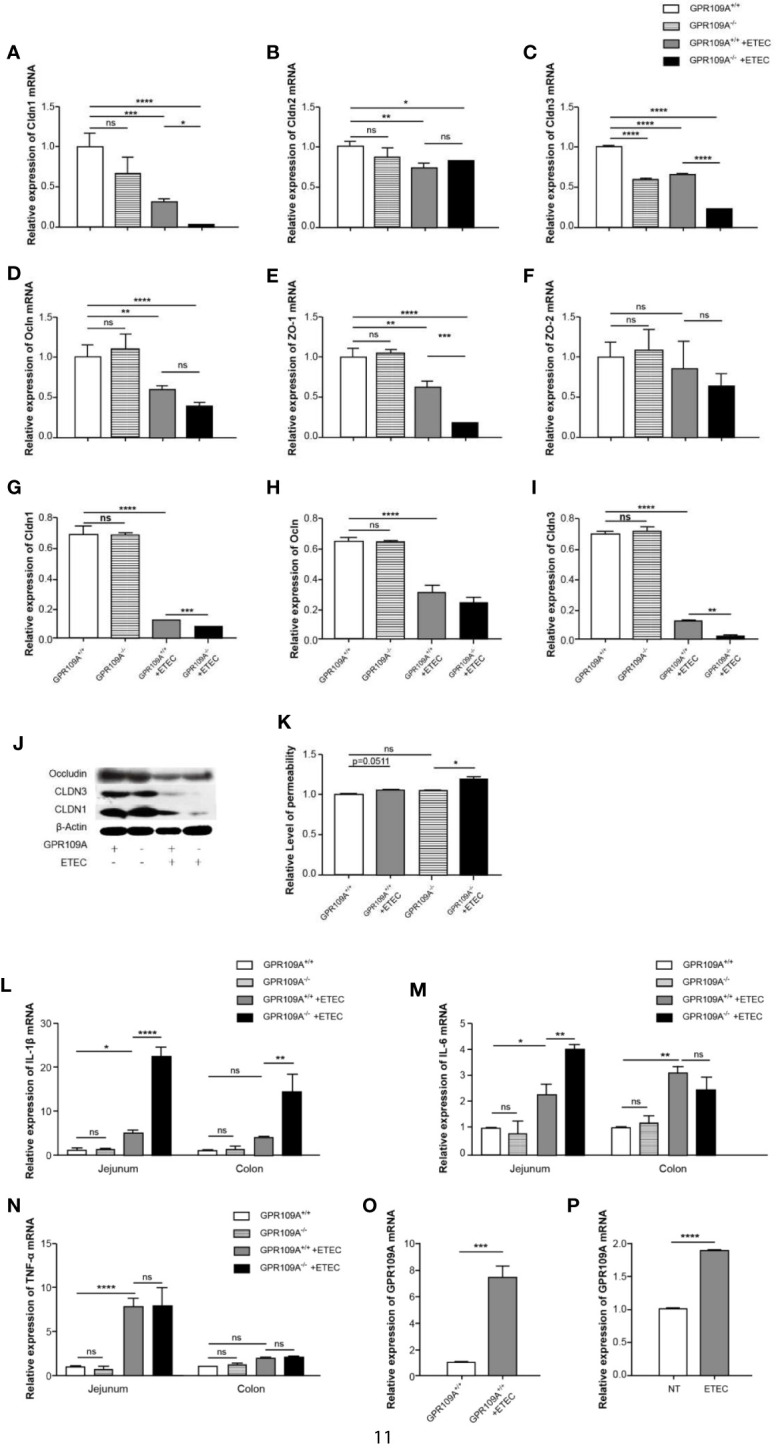
GPR109A deficiency showing in the increased intestinal permeability and production of proinflammatory mediators following ETEC mucosal infection. On day 5 post infection, the expression of tight junction genes in the jejunum and proinflammatory mediators in the jejunum and colon was examined. **(A–F)** Expression of Cldn1, Cldn2, Cldn3, Ocln, Zo-1, and Zo-2 by qPCR (*n* = 3). **(G–J)** Representative immunoblots **(J)** and quantification **(G–I)** of Cldn1, Cldn3, and Ocln expression in the jejunum (n = 3). **(K)** Relative permeability of FITC-dextran (40KD) by oral administration (*n* = 3). **(L–N)** Expression of IL-1β, IL-6, and TNF-α mRNA in the jejunum and colon tissue homogenate from the mice receiving different treatments (*n* = 3). **(O)** Expression of GPR109A mRNA in the jejunum tissue homogenate from GPR109A^+/+^ mice after infection (*n* = 3). **(P)** Expression level of GPR109A mRNA in Caco-2 cells with the challenge of ETEC. The cells were inoculated with ETEC (5×10^7^ CFU/well, MoI = 100) in the antibiotic-free medium for 1 h. The values are expressed as mean ± standard deviation (SD). ns means no significance, ^*^ *p* < 0.05, ^**^ *p*< 0.01, ^***^ *p* < 0.001, ^****^ *p* < 0.0001. The presented data are the average of three independent experiments.

The intestinal mucosa acts as a physiological barrier of the digestive tract that protects against microbes, toxins, and antigens. To investigate changes in intestinal permeability following ETEC infection in mice and to further explore the mechanism underlying the function of GPR109A in controlling intestinal permeability, we evaluated the level of transcription of different tight junction genes. The RT-PCR results showed that ETEC infection reduced the expression of intestinal tight junction genes. Furthermore, we did not observe any difference between WT and GPR109A knockout mice neither in the expression of tight junction proteins nor in the levels of inflammatory cytokines before ETEC gavage, except for Cldn3. Compared with the GPR109A^+/+^ mice, the expression of Cldn1, Cldn3, and Zo-1 was reduced in GPR109A^–/–^ mice after the ETEC challenge, whereas there was no difference in the expression of Cldn2, Ocln, and Zo-2 (**Figures 2A–F**). Western blotting analysis further confirmed the reduced expression of Cldn1 and Cldn3 following ETEC infection in the GPR109A^–/–^ mice compared to the GPR109A^+/+^ mice (**Figures 2G–J**). We determined the intestinal permeability of FITC-dextran (40 kD) in the WT and GPR109A^–/–^ mice challenged by ETEC (**Figures 2K**). The results showed that ETEC significantly increased the intestinal permeability in GPR109A^–/–^ mice, indicating that the intestinal barrier was disrupted by ETEC gavage in the absence of GPR109A.

In order to further prove that GPR109A maintained intestinal homeostasis, we assessed the levels of proinflammatory cytokines in the jejunum and colon of the ETEC-infected GPR109A^+/+^ and GPR109A^–/–^ mice. Although there was no obvious change in TNF-α (**Figure 2N**) between the GPR109A^+/+^ and GPR109A^–/–^mice, when compared with the GPR109A^+/+^ mice, the GPR109A^–/–^mice had significantly higher levels of IL-1β, IL-6 in the jejunum and higher levels of IL-1β in the colon after ETEC gavage (**Figures 2L–M**). These results indicated that GPR109A not only reduced the inflammation invoked by ETEC in the intestine but also preserved the intestinal integrity under ETEC infection.

### Activated GPR109A Affects IgA-Related Gene Expression

**Figure 3:**
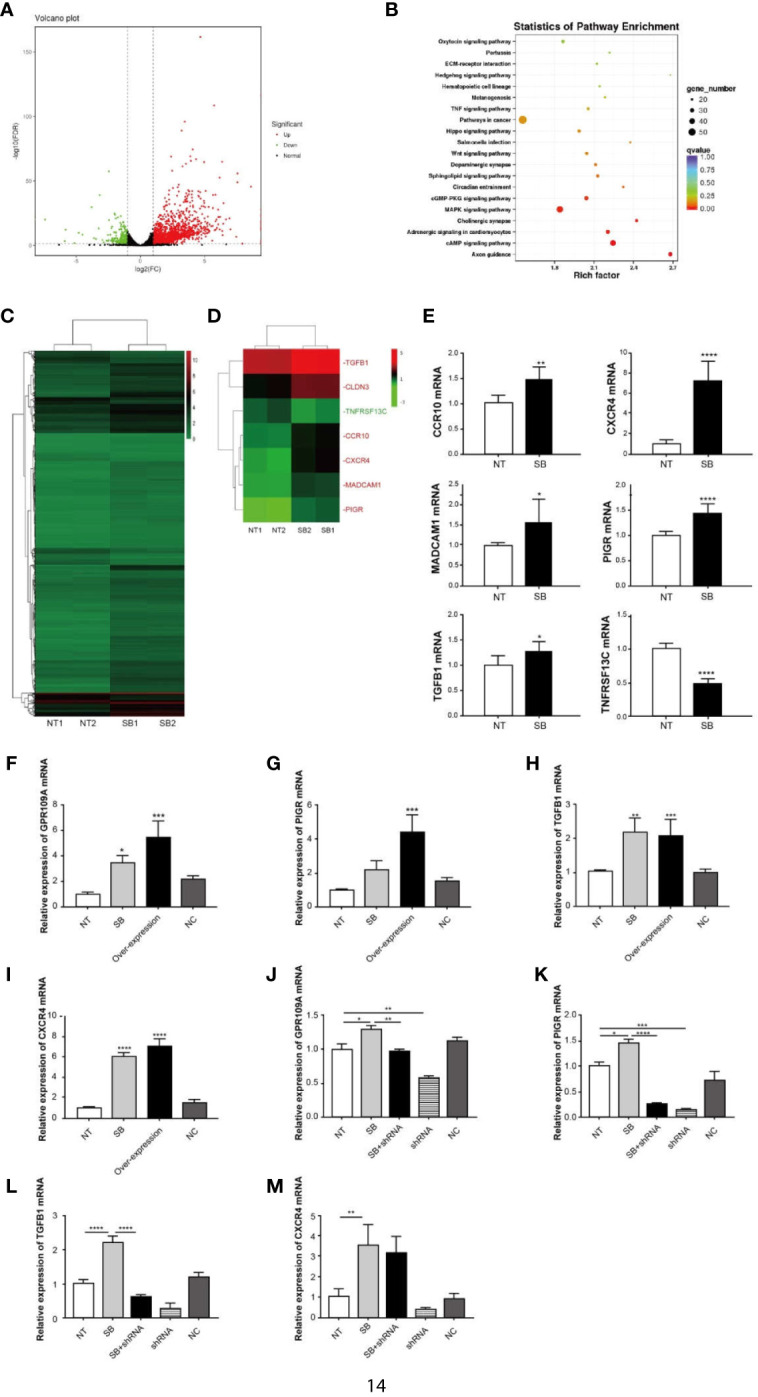
GPR109A receptor affects IgA-related gene expression. Total RNA was isolated from Caco-2 cells following treatment with 2 mM sodium butyrate (SB). High-throughput sequencing was performed, followed by bioinformatics analysis. **(A)** Volcano diagram of differentially expressed genes between NT and sodium butyrate treatment (*n* = 2). Genes with *p*-values < 0.05 and an absolute value of log2 (fold change)>1 were considered to be differentially expressed. The red dots indicate upregulated genes and the green dots indicate down-regulated genes. **(B)** KEGG enrichment of differentially expressed genes (*n* = 2). **(C)** Heatmap of differentially expressed genes (*p* < 0.05) (*n* = 2). **(D)** Heatmap of IgA production-related genes, expression of which was altered by SB (*p* < 0.05). The red text refers to upregulation and the green text refers to downregulation (*n* = 2). **(E)** q-PCR analysis of differentially expressed genes in the two groups (*n* = 3). **(F)** Overexpression of GPR109A plasmid increased the expression of GPR109A mRNA in Caco-2 cells, but the noncoding (NC) plasmid failed to increase the levels of GPR109A mRNA (n = 3). **(G–I)** Expression of PIGR, TGFB1, and CXCR4 was detected by q-PCR after overexpressing GPR109A (*n* = 3). **(J)** shRNA lentivirus was used to knock down the GPR109A. shRNA lentivirus reduced the expression of GPR109A mRNA in Caco-2 cells, but the NC failed to decrease the levels of GPR109A mRNA (n = 3). **(K–M)** Expression of PIGR, TGFB1, and CXCR4 was detected by q-PCR after knocking down GPR109A by shRNA (*n* = 3). NT indicates no treatment, shRNA represents NT + shRNA, NC indicates noncoding plasmid or lentivirus. Values are expressed as mean ± standard deviation (SD). ^*^ *p* < 0.05, ^**^ *p* < 0.01, ^***^ *p* < 0.001, ^****^ *p* < 0.0001. A representative of 3 independent experiments is shown.

An elevation in the expression of GPR109A in the intestinal tissue was observed following the ETEC infection (**Figure 2O**). Similar results were reproduced in Caco-2 cells, which were inoculated with ETEC *in vitro* (**Figure 2P**). To explore the function of GPR109A in intestinal protection, the total RNA was isolated from Caco-2 cells, following the treatment with 2 mM sodium butyrate, a GPR109A receptor agonist. Caco-2 cells represent an ideal model of colonic epithelial cells. Afterward, high-throughput sequencing was performed and the results were subjected to bioinformatics analysis. Sodium butyrate altered the expression of 1,958 genes (*p* < 0.05) in Caco-2 cells, with 1,673 transcripts significantly upregulated and 285 significantly downregulated (**Figures 3A, C**). A statistical analysis of the enriched pathways (**Figure 3B**) revealed that the different genes induced by sodium butyrate activated GPR109A were mainly enriched in *Salmonella* infection. The MAPK signaling pathway and TNF signaling pathway could be involved in inhibiting the ETEC infection and inflammation. Among the differentially expressed genes (DEGs), we screened six genes, namely CXCR4, TGFB1, MADCAM1, CCR10, PIGR, and TNFRSF13C, which are involved in the intestinal immune network for IgA production (**Figure 3D**). These genes were selected for validation by q-PCR (**Figure 3E**). To test whether sodium butyrate affected the expression of these genes by activating GPR109A, colon epithelial cells were transfected with a GPR109A-overexpressing plasmid (**Figure 3F**). The expression of PIGR, TGFB1, and CXCR4 increased with an increase in the expression of GPR109A (**Figures 3G–I**). Subsequently, the lentivirus-mediated knockdown of GPR109A mRNA in the cells (**Figure 3J**) reduced the expression of PIGR and TGFB1 (**Figures 3K, L**); however, the expression of CXCR4 was not affected by the overexpressing GPR109A (**Figure 3M**). TGFβ1 mediates the effect of retinoic acid (RA) by enhancing the production of IgA by B cells (21) and PIGR facilitates the transfer and secretion of IgA from the intestinal lamina propria (LP) to the intestinal lumen (22). These results revealed that GPR109A regulated the production of IgA-related genes.

### GPR109A Regulates Intestinal SIgA Production Under ETEC Infection

**Figure 4:**
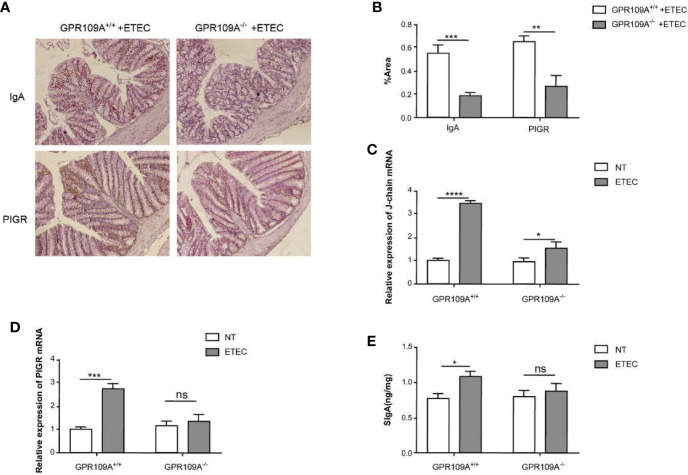
GPR109A receptor regulates intestinal SIgA production. **(A, B)** Representative images. The expression of SIgA and PIGR proteins after ETEC infection was quantified by immunohistochemical analysis (*n* = 6). **(C, D)** The expression of J-chain and PIGR mRNA was detected by qPCR in the colon tissue homogenate (*n* = 3). **(E)** SIgA contents in the jejunum were analyzed by ELISA kits (*n* = 3). Values are expressed as mean ± standard deviation (SD). ns means no significance, ^*^ *p* < 0.05, ^**^ *p* < 0.01, ^***^ *p* < 0.001, ^****^ *p* < 0.0001. The data shown are the average of three independent experiments.

IgA^+^ B cells enter blood circulation from the submucosal lymphatic vessels, following antigen stimulation. They subsequently differentiate, proliferate, and spread into the submucosal LP to become mature IgA plasma cells. Plasma cells first synthesize J chain and dimer IgA in the cytoplasm and then combine with PIGR on the inner surface of epithelial cells. They transport these molecules to the surface of epithelial cells through PIGR and form SIgA, and finally release them (23). To ascertain the effects of GPR109A on intestinal SIgA, diaminobenzidine (DAB) staining was used to assess the distribution of IgA^+^ cells and PIGR protein in the colon. The levels of SIgA were assessed by ELISA in the jejunum homogenates. Real-time PCR was performed to detect the expression of mRNA of J-chain and PIGR. As expected, compared with GPR109A^–/–^mice, more IgA^+^ cells and PIGR proteins were distributed in the intestinal epithelium of GPR109A^+/+^ mice following ETEC infection (**Figures 4A, B**). The expression of J-chain in both GPR109A^+/+^ and GPR109A^–/–^mice was significantly increased (**Figure 4C**), whereas that of PIGR was only increased in GPR109A^+/+^ mice (**Figure 4D**). Accordingly, the levels of SIgA in the jejunum homogenate of GPR109A^+/+^ mice were higher than in GPR109A^–/–^mice (**Figure 4E**). Overall, these results proved that GPR109A is required for the transfer of IgA to the intestinal lumen under ETEC infection.

### Butyrate Prevents Colonization and Translocation of ETEC in the Intestine in a GPR109A-Dependent Manner

**Figure 5:**
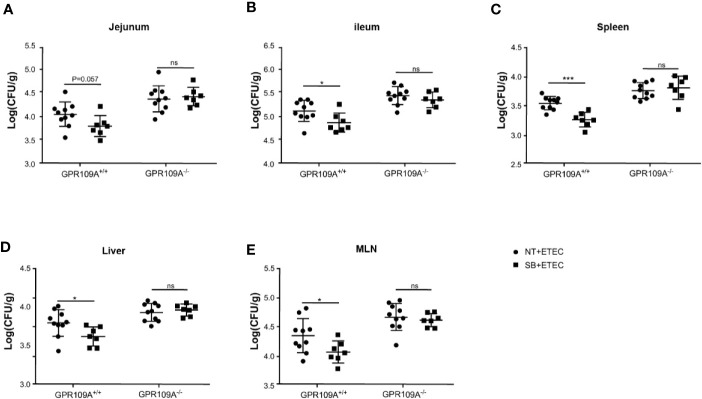
Sodium butyrate limits the colonization and translocation of ETEC in GPR109A^+/+^ mice. GPR109A^+/+^ and GPR109A^–/–^ mice were treated with streptomycin before oral administration of 1× 10^9^ colony-forming units (CFUs) of ETEC for five days. Sodium butyrate treatment was started one day before the addition of antibiotics and continued throughout the protocol. Bacterial load in **(A)** jejunum, **(B)** ileum, **(C)** spleen, **(D)** liver, and **(E)** mesenteric lymph nodes (MLN) is shown. SB: sodium butyrate, (*n* = 7). Values are expressed as mean ± standard deviation (SD), ns means no significance, ^*^ *p* < 0.05, ^***^ *p* < 0.001. The data shown are the average of three independent experiments.

Butyric acid acts as an exogenous ligand to activate GPR109A. To further examine the relationship between GPR109A and ETEC infection and whether sodium butyrate affected the colonization and translocation of ETEC by activating GPR109A, we added 5 g/L sodium butyrate to the drinking water of mice. The results showed that sodium butyrate significantly inhibited the colonization of ETEC in the jejunum (*p* = 0.057; **Figure 5A**) and ileum (**Figure 5B**) of GPR109A^+/+^ mice. Moreover, it significantly reduced the ETEC load in the spleen, liver, and MLNs (**Figures 5C–E**). However, it did not exert a protective effect on GPR109A^–/–^ mice.

**Figure 6:**
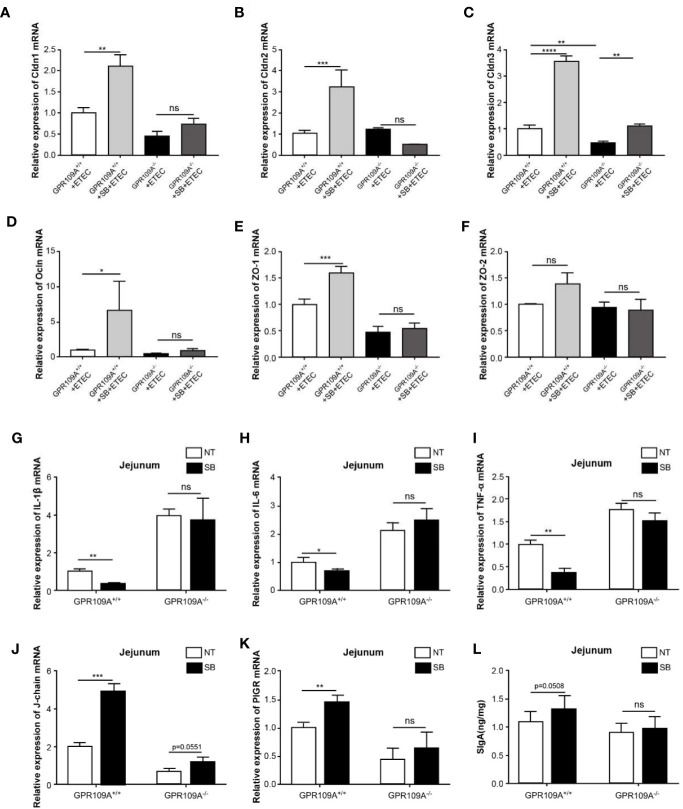
Sodium butyrate maintains gut homeostasis in a GPR109A-dependent manner during ETEC infection. GPR109A^+/+^ and GPR109A^–/–^mice were treated with streptomycin before the oral administration of 1× 10^9^ colony-forming units (CFUs) of ETEC for five days. Sodium butyrate treatment was started one day before the addition of antibiotics and continued throughout the protocol. The expression of tight junction proteins **(A)** Cldn1, **(B)** Cldn2, **(C)** Cldn3, **(D)** Ocln, **(E)** Zo-1, and **(F)** Zo-2 was examined by q-PCR (*n* = 3). **(G–J)** Expression of IL-1β, IL-6, and TNF-α mRNA in the jejunum tissue homogenate obtained from mice with different treatments (*n* = 3). **(K, L)** Expression of J-chain and PIGR mRNA in the jejunum tissue homogenate (*n* = 3). (N) SIgA contents in the jejunum were analyzed by ELISA kits (*n* = 3). SB: sodium butyrate, (*n* = 3). Values are expressed as mean ± standard deviation (SD), ns means no significance, ^*^ *p* < 0.05, ^**^ *p* < 0.01, ^***^ *p* < 0.001, ^****^ *p* < 0.0001. Data are based on an average of three independent experiments.

To investigate the function of sodium butyrate in preserving the intestinal barrier integrity of infected mice, we detected the transcription levels of tight junction genes. Sodium butyrate reversed the decrease in the expression of tight junction proteins Cldn1, Cldn2, Cldn3, Ocln, and Zo-1 caused by ETEC infection in GPR109A^+/+^ mice, but not in GPR109A^–/–^mice (**Figures 6A–F**). Similarly, sodium butyrate reversed the increased expression of proinflammatory cytokines IL-1β, IL-6, and TNF-α in GPR109A^+/+^ mice but showed no reversal effect in GPR109A^–/–^mice (**Figures 6G–I**). Sodium butyrate significantly increased the expression of J-chain and PIGR in GPR109A^+/+^ mice (**Figures 6J–K**), increasing the secretion of IgA in GPR109A^+/+^ mice (*p* = 0.0508) but not in GPR109A^–/–^mice (**Figure 6L**). These data suggested that sodium butyrate reduced the colonization and translocation of ETEC, improved intestinal permeability, inhibited intestinal inflammation, and promoted the secretion of IgA through the GPR109A receptor. Thus, the protective effect of sodium butyrate on intestinal mucosa was dependent on the GPR109A receptor.

## Discussion

After activation by its ligands, GPR109A can significantly inhibit the body’s inflammatory response in different diseases such as atherosclerosis, obesity, sepsis, diabetes, colitis, and neurodegenerative diseases (10). However, the function of GPR109A during ETEC infection and its underlying mechanism remains unknown. To explore the underlying mechanisms, we treated mice *via* oral gavage of the pathogenic bacteria ETEC and found that GPR109A-deficient mice were more susceptible to ETEC, which was manifested by enhanced intestinal colonization and wider systemic transmission. This observation demonstrated the significance of GPR109A in maintaining intestinal homeostasis during ETEC infection.

ETEC is a widespread pathogen, causing diarrhea in humans and young animals. After entering the small intestine through the digestive system, ETEC attaches to the intestinal epithelial cells *via* the adhesin (fixation factor) present on the pili. Colonized ETEC produces enterotoxins that damage epithelial cells in the attachment zone, resulting in the spread of the pathogen and colonization of systemic sites (5). Consequently, the more severe ETEC colonization and translocation observed in GPR109A^–/–^ mice could be attributed to the damaged intestinal barrier and inability of the intestinal tract to remove foreign bodies.

The gut is an important part of the digestive system. The integrity of the intestinal mucosal barrier needs to be preserved to maintain a normal intestinal function. When the permeability of the mucosa increases to a certain extent, certain macromolecular substances in the intestine, such as bacteria and toxins, enter the peripheral tissues through the damaged intestinal mucosa, causing bacterial translocation to the liver, lymph, and blood, leading to gut origin infection and even multiple organ failure (MOF) (24). Tight junction proteins maintain the balance between intestinal epithelial structure and function and act as the main barrier against foreign antigens, microorganisms, and other foreign bodies. The functional and structural loss of tight junction protein has been reported in multiple intestinal diseases, such as inflammatory bowel disease (IBD) and diarrhea (13, 17). Microorganisms disrupt the intestinal tight junctions to facilitate their spread in the host. Therefore, we examined the effect of GPR109A on the expression of tight junction proteins. Our results indicated that the levels of cldn1, cldn3, Ocln, and Zo-1 in GPR109A^–/–^mice were significantly reduced compared with those in GPR109A^+/+^ mice after ETEC infection. The destruction of intestinal wall integrity increased the susceptibility of GPR109A^–/–^mice to pathogen invasion, which was manifested by higher bacterial spread and intestinal inflammation.

The dysregulation of the intestinal immune response is involved in the development of IBD (25). Intestinal inflammatory response and immune response can be closely monitored and regulated by several physiological mechanisms to maintain intestinal immune homeostasis (26, 27). To investigate whether GPR109A regulated local immune responses in the gastrointestinal tract, a model of enteritis was established by oral administration of 1 × 10^9^ CFUs of ETEC. The data demonstrated that GPR109A^–/–^mice were more sensitive to ETEC mucosal infections and had a higher proinflammatory mediator production than GPR109A^+/+^ mice, causing greater damage to the intestinal tract. Excessive inflammatory response and intestinal damage in GPR109A^–/–^ mice indicated its function in restricting inflammatory responses and maintaining intestinal immune homeostasis. In addition, earlier studies have reported that proinflammatory factors increased permeability and decreased the expression of the tight junction molecule Zo-1 in intestinal cell line Caco-2 (28).

ETEC has been demonstrated to induce cell death and decrease the expression of tight junction proteins in intestinal epithelial cells (29–31) and a mouse model (32). In this experiment, we also found that ETEC significantly decreased the expression of tight junction proteins. Furthermore, GPR109A^–/–^ mice showed a drastic decrease in cldn1, cldn3, Ocln, and ZO-1. Meanwhile, ETEC invoked proinflammatory cytokine expression in the mucosa and GPR109A^–/–^ mice showed the highest expression of IL-1β and IL-6. Sodium butyrate significantly protected GPR109A^+/+^ mice from decreasing the expression of tight junction proteins under the ETEC challenge. The protective effect of sodium butyrate was lost in the absence of GPR109A. Along with the results of the Caco-2 cells, we confirmed that the activated GPR109A could not directly regulate the expression of tight junction proteins other than cldn3. A reasonable explanation is that activated GPR109A was involved in limiting inflammatory cytokines, which protected epithelial cells from ETEC by facilitating the expression of tight junction proteins. It has been reported that IL-1 β and IL-6 can reduce the expression of tight junction proteins (33); thus their inhibition maintains the expression of tight junction protein (34). Therefore, the inhibition of GPR109A on inflammatory response can be regarded as a potential mechanism to maintain the integrity of the barrier. An alternative mechanism is that ETEC promotes apoptosis in epithelial cells (35, 36). However, the activation of GPR109A or sodium butyrate treatment can effectively inhibit the apoptosis of epithelial cells (37). This also explains the phenomenon we observed *in vivo*, that the increased expression of tight junction proteins in GPR109A mice compared to the knockout mice after butyric acid treatment was due to the protective effects of GPR109A in preventing cell death. However, how GPR109A affects the expression of tight junction proteins during ETEC infection needs to be explored further.

Secretory IgA is the first line of defense on the mucosal surface of the intestine. It prevents the adhesion of intestinal microbes to the surface of the intestinal mucosa, neutralizes the toxins produced by the bacteria, and exerts an antibacterial effect in combination with the complement system (38–40). The dysfunction of intestinal SIgA secretion reduced the intestinal colonization resistance and promoted intestinal bacterial translocation (41). Our results indicated the involvement of GPR109A in the intestinal immune network for IgA production. Compared with the GPR109A^–/–^mice, the GPR109A^+/+^ mice produced more SIgA after ETEC infection. A recent study reported that sodium butyrate promoted the expression of integrin avβ8 in dendritic cells (DCs) and increased the number of IgA^+^ cells (41), indicating that it promoted the IgA response in the colon in a T cell-independent manner. Virdi et al. (42) found that the addition of monomeric IgA protected the piglet model from ETEC infection. Similarly, Liu et al. (43) reported that l-glutamine and l-arginine promoted intestinal IgA secretion and protected against ETEC infection in mice. We reasonably hypothesized that GPR109A exerts its protective effects against ETEC infection in mice by promoting IgA secretion.

Some recent studies have shown that sodium butyrate relieves enteritis caused by several pathogens, such as *Salmonella enterica* serovar Typhimurium (44), *Shigella* (45), and *Clostridium difficile* (16) *via* different molecular mechanisms, such as affecting the factors involved in bacterial colonization and the direct or indirect production of toxins (44), stimulating host’s intestinal immune response (45), and protecting IECs directly (16). We showed that sodium butyrate effectively protected the intestinal mucosa from ETEC. Butyric acid protects the intestinal barrier *via* multiple mechanisms. It supplies energy to intestinal epithelial cells, improves the integrity of the intestinal mucosa by promoting its proliferation and differentiation, and prevents virulence factors and other exogenous substances from entering the bloodstream and preventing inflammation (46). Butyrate is a histone deacetylase inhibitor that reduces inflammation by activating the AP-1 pathway of the intestinal tract epithelial (47), downregulating the expression of proinflammatory cytokines, and increasing the level of Fas protein to induce T cell apoptosis (48). Kelly et al. (49) reported that butyrate increased the stability of HIF-1, which is closely related to the health of the intestinal barrier, by controlling the expression of genes associated with inflammation and apoptosis in the IEC *via* regulating metabolism and oxygen consumption. Another previous study also reported that butyrate was produced by commensal bacteria that promote T-cell-independent IgA class switching recombination (CSR) in the mouse colon (50). Although we cannot exclude the contribution of these mechanisms, which are induced by butyrate in mitigating ETEC infection, our data suggested that GPR109A is relevant in this protection. Data indicated that butyric acid was not involved in exerting protective effects in the ETEC infection in GPR109A^–/–^mice, which is manifested by no significant change in the levels of inflammatory factors, intestinal permeability, bacterial colonization, and translocation of GPR109A^–/–^mice after the addition of sodium butyrate. Moreover, the addition of sodium butyrate increased the secretion of IgA in the intestinal tract of GPR109A^+/+^ mice; however, this effect was not observed in GPR109A^–/–^mice.

GPR109A has been reported to limit colonic inflammation. Singh et al. (51) observed that GPR109A deficiency enhanced the susceptibility to lethal colitis and activation of GPR109A in immune cells, and colonic tissue was necessary for suppressing colonic inflammation. Our previous study (13) also indicated that GPR109A mediated the function of sodium butyrate in inhibiting inflammation and protecting the intestinal epithelial barrier in the TNBS-induced IBD mouse model by inhibiting the phosphorylation of AKT and NF-Kb p65 signaling pathways. Feng et al. (17) reported that sodium butyrate increased the expression of Cldn3 in the colon *via* the Akt signaling pathway in a GPR109A-dependent mechanism. Similarly, Chen et al. (14) reported that GPR109A improved the survival rate, reduced the level of proinflammatory cytokines, and decreased the intestinal permeability in a CLP-induced mouse sepsis model. The mechanism could be associated with the regulation of the gut flora by the receptor. Our study extended these findings by confirming that sodium butyrate enhanced the resistance of IECs to ETEC in a GPR109A-dependent manner.

**Figure 7:**
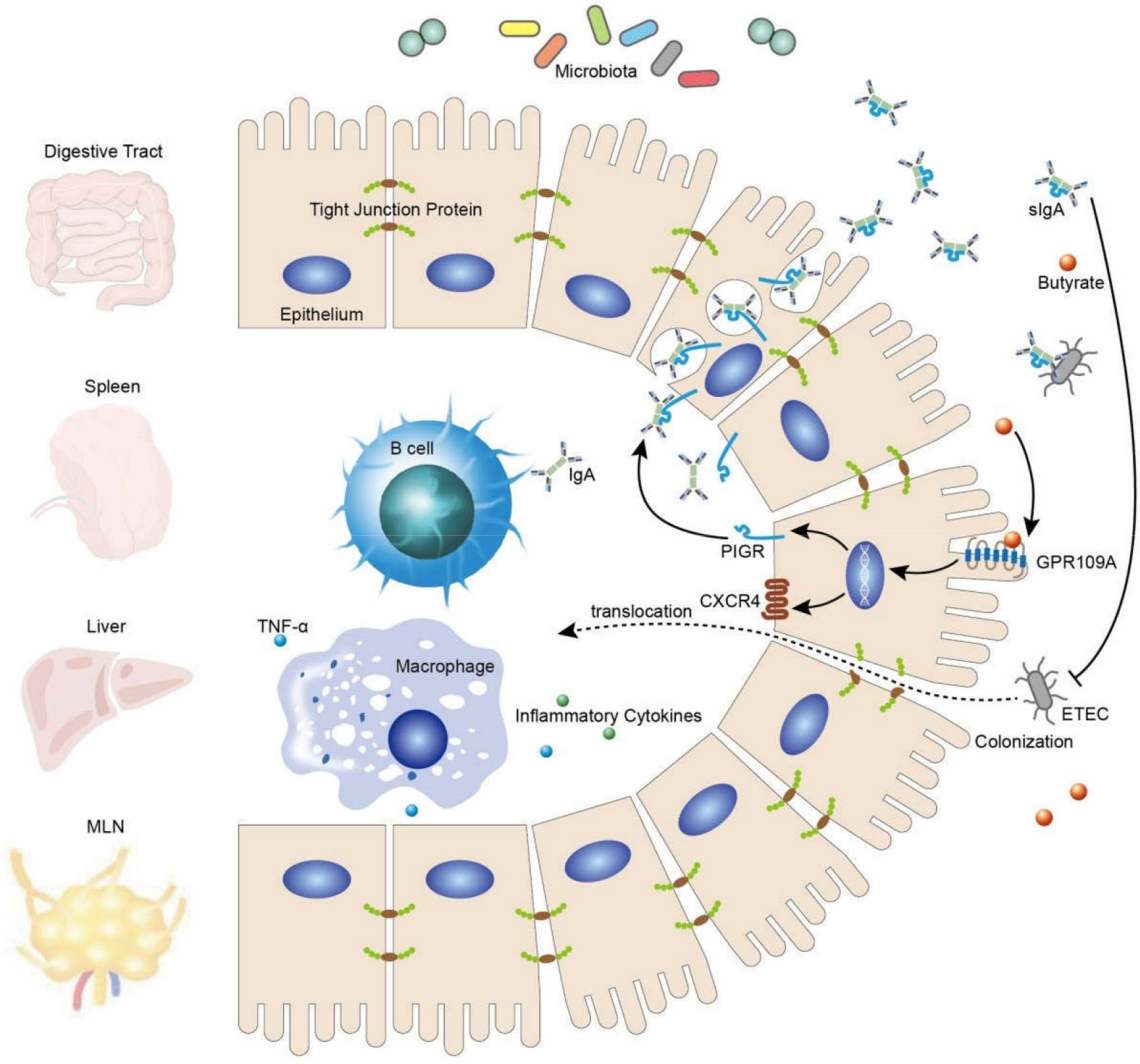
Schematic representation of the proposed mechanism.

In summary, our findings confirmed that activated GPR109A effectively protected the gut against the colonization and translocation of ETEC and maintained the integrity of the intestinal barrier, possibly by promoting the secretion of intestinal IgA (**Figure 7**).

GPR109A is essential for mediating the effect of butyrate in epithelium cells by promoting the expression of PIGR and facilitating the secretion of IgA, thus inhibiting ETEC colonization and translocation and, consequently, maintaining intestinal barrier integrity.

## Data Availability Statement

The original contributions presented in the study are publicly available. This data can be found here: https://www.ncbi.nlm.nih.gov/sra/SRX9596427.

## Ethics Statement

The animal study was reviewed and approved by Institutional Animal Care and Use Committee of Zhku.

## Author Contributions

WW, YH, and YZ designed the research. WW, YG, BY, XJ, and YL conducted research. YG and BY undertook model building and sample extraction. YG, BY, XJ, and GY undertook immunohistochemical and ELISA analyses. GY, JL, XJ, and ML performed vector construction. YG, JL, and ML investigated the molecular mechanism, including RT-PCR and western blot. WW, YZ, and YG analyzed data. WW, YG, and YZ wrote the manuscript. WW approved and checked the content of the final published manuscript. All authors contributed to the article and approved the submitted version.

## Funding

This work was supported by the National Natural Science Foundation of China (31872442) and Guangdong Science and Technology Program Project (2017A030303022).

## Conflict of Interest

The authors declare that the research was conducted in the absence of any commercial or financial relationships that could be construed as a potential conflict of interest.
