# Supplementary material for: G Protein-Coupled Receptor 109A Maintains the Intestinal Integrity and Protects Against ETEC Mucosal Infection by Promoting IgA Secretion
Source: Front Immunol. 2021 Jan 8;11:583652. doi: 10.3389/fimmu.2020.583652 (PMC7821714; doi:10.3389/fimmu.2020.583652)
Supplement: Supplementary file 1 [file DataSheet_1.pdf]

Supplementary materials

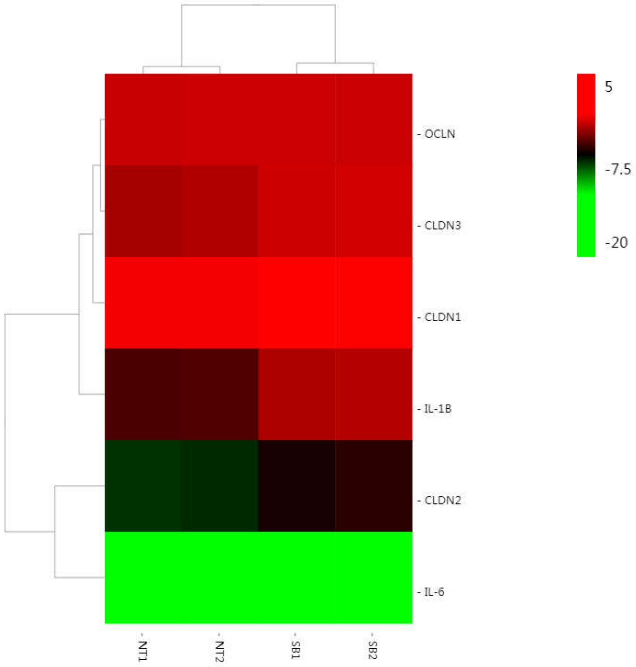

Supplementary fig-1 heat map for tight junction protein expression (FPKM) from Caco-2 treated with sodium butyrate.

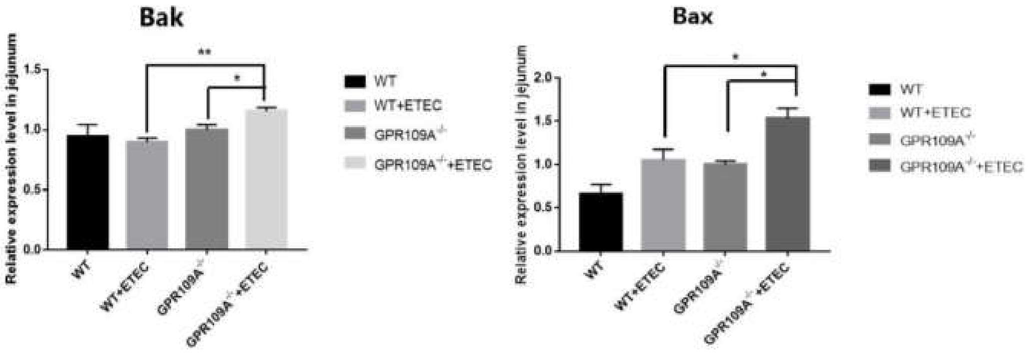

Supplementary fig-2 expression level of apoptosis related genes Bak and Bax by qPCR in scraped intestinal mucosa cells with the existence or absence of GPR109A under ETEC challenge.

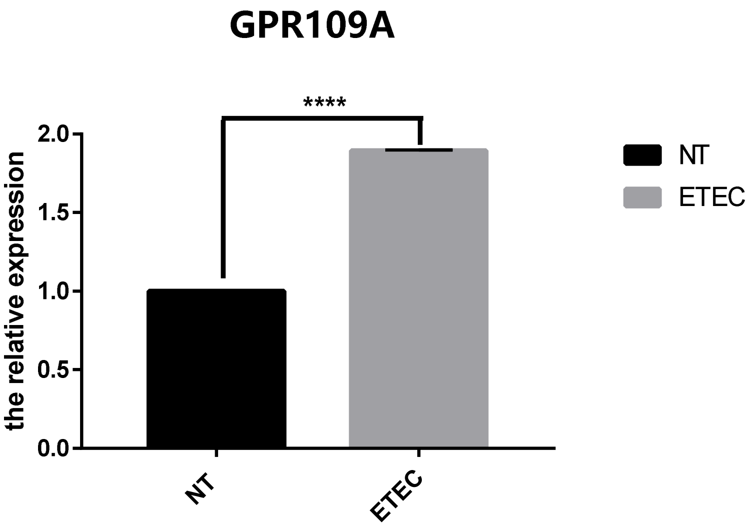

Supplementary fig-3. The expression of GPR109A in Caco-2 cells. Cells were inoculated with ETEC (5×10^7 CFU/well, MoI=100) in antibiotic-free medium and incubated for 1 h.
